# Tooth wear in patients treated with HIV anti-retroviral therapy

**DOI:** 10.1186/s12903-019-0818-1

**Published:** 2019-06-26

**Authors:** Harjit Singh Sehgal, Richie Kohli, Edward Pham, Grace E. Beck, Jay R. Anderson

**Affiliations:** 1 Department of Periodontology, School of Dentistry, Oregon Health & Science University, 5N034 SDPERI, 2730 SW Moody Ave, Portland, Oregon 97201-5042 USA; 2 Department of Community Dentistry, School of Dentistry, Oregon Health & Science University, Portland, OR USA; 3 School of Dentistry, Oregon Health & Science University, Portland, OR USA; 4 Department of Endodontics, The University of Pennsylvania School of Dental Medicine, Philadelphia, PA USA; 5 Clinica Sierra Vista, Bakersfield, CA USA

**Keywords:** Tooth wear, Bruxism, Community dentistry, Dental, Anti-retroviral therapy

## Abstract

**Background:**

The objective of this study was to elucidate the relationship between HIV anti-retroviral therapy and tooth wear.

**Methods:**

Assessment of tooth wear was conducted both with a survey questionnaire and clinical assessment at Russell Street Dental Clinic in Portland, Oregon. The survey questionnaire comprised of questions on study participant’s gender, age, HIV status, current medications, awareness of tooth grinding or clenching, jaw soreness, tooth or gum soreness, and frequency of headaches. For the clinical evaluation, a dental provider recorded the degree of wear on each tooth using a scale of 0–3. An individual tooth-wear index was used to rank patients with regard to incisal and occlusal wear. Data analysis included descriptive analysis, tests of association and regression analysis using SPSS V.24.

**Results:**

The study sample involved 93 patients (HIV + ve = 60, HIV–ve = 33) with age range of 20-90 yrs. (mean = 49 yrs., s.d = 13.3). 92 and 67% participants of the HIV + ve and HIV-ve groups, respectively, presented with tooth wear. The mean tooth wear index was higher in HIV + ve patients than HIV–ve patients (8.2 vs. 7.8), however, this difference was not statistically significant (*p* > 0.05). A significant, positive correlation was found between HIV presence and tooth wear index, after accounting for age (B = 0.71, *p* < 0.05). The number of years on anti-retroviral therapy alone was positively correlated with tooth wear index (R^2^ = 0.116, p < 0.05). After controlling for age, years of anti-retroviral therapy use was positively correlated with tooth wear index (B = 0.047, *p* > 0.05).

**Conclusions:**

The findings from this study suggest that HIV + ve patients, who are on anti-retroviral therapy have significant tooth wear, although more studies with larger sample size are needed to confirm this. There is a critical need to initiate a dialogue with medical providers about tooth wear as a possible side effect of antiretroviral therapy and to introduce appropriate preventive measures.

## Background

People living with human immunodeficiency virus (HIV) and acquired immunodeficiency syndrome (AIDS) (PLWHA) face tremendous problems regarding their oral health. PLWHA have an increased incidence of poor oral health, which further exacerbates their other medical conditions. PLWHA have reported high unmet oral health needs and low utilization of oral health services [8, 19]. Furthermore, PLWHA face many barriers to acquiring oral health care, including lack of dental insurance, limited financial resources [8], shrinking adult dental Medicaid services [19], and perceived stigma within health care systems [28, 29].

Clinical observation has suggested that many PLWHA are diagnosed with bruxism. Bruxism is characterized by the involuntary clenching or grinding of the teeth especially during sleep and can cause severe oral health problems, including the destruction of tooth structure, temporomandibular joint dysfunction, myofascial pain, and severe sleep disturbances [13, 14, 17, 30]. Bruxism has been reported to occur about two times more frequently in PLWHA than in the general population. In a systematic review of 26 papers, researchers found two articles stating bruxism has a prevalence of up to 31% of adults [18]. In a study conducted by Juvino et al. in 2017, 64% of HIV/AIDS participants presented with bruxism.

Not only are AIDS patients susceptible to numerous oral and systemic ailments, but also these individuals are faced with psychological challenges. PLWHA are more likely to develop depression and anxiety than the general population ([2]; Sun, Wu, Ou, Lu, & Wang, 2014). Both depression and anxiety affect a person’s quality of life [5, 34] as well as their ability to follow the treatment regimen for HIV and attend to their oral self-care [3, 15, 25]. The presence of psychological disorders is known to result in various oral health problems, such as bruxism [6, 21] and tooth wear [23]. Several researchers found bruxers to have increased incidence of anxiety than non-bruxers [12, 16]. While currently available depression and anxiety treatments are generally well tolerated and safe, a class of medications used to treat depression, selective serotonin reuptake inhibitors (SSRIs), increases teeth grinding and therefore bruxism [26]. The combination of HIV, medications for HIV treatment, and the impact of SSRI may lead to progressively severe dental attrition.

The antiretroviral medications that PLWHA depend on have negative effects on their oral health. Many oral health issues experienced by PLWHA are related to the side effects of the medications themselves, including xerostomia (or dry mouth) which is a significant complication of HIV and/or side effect of ART medications [27]. In addition, antiretroviral therapy can have various side effects including mood and sleeping disturbances [1]. The combination of insomnia and medication side effects increases the likelihood of clenching and grinding of PLWHA patients. HIV drugs in combination with other medications that can cause dry mouth [33], clenching and grinding (A.K. Johansson, Omar, Carlsson, & A. Johansson, 2012) leave teeth surfaces vulnerable to both mechanical wear and chemical wear [24, 31].

Antiretroviral therapy can have various other side effects including weight gain, weight loss, bone and bleeding problems, as well as an increased risk of heart disease, kidney and/or liver problems (Johansson, 2012). However, not many studies exist regarding oral health and parafunctional habits associated with medications used to treat AIDS. Those studies that do exist examine the oral health pathologies experienced as a result of opportunistic infections, rather than the therapeutic regimens [20]. Due to the lack of research, this topic is not well understood and little is known about the effects of antiretroviral therapy on oral health. The present study is among the few in the literature and the first in Oregon to establish an association between tooth wear and the antiretroviral medications taken by HIV positive patients.

## Methods

This study protocol was approved by the Oregon Health & Science University Institutional Review Board (IRB00010706).

### Study design

This is a cross-sectional study that utilizes survey and clinical assessment of dentition among HIV positive patients on antiretroviral therapy. Participants were recruited from the Russell Street Clinic, a walk-in, community dental clinic in Portland, Oregon. Patients were screened by survey. Surveys asked participants to self-report whether they have symptoms of bruxism. Additional subject characteristics were documented. A dentist conducted clinical evaluations. Following the study, informational brochures regarding the side effects of HIV treatment on oral health were developed and distributed among patients at the clinic.

### Sample

At the Russell Street Clinic, patients were screened based on eligibility criteria as follows: at least 18 years of age, not pregnant, have at least 25 natural permanent teeth, HIV positive (HIV + ve) and on highly active antiretroviral therapy (HAART), or HIV negative (HIV-ve). If the patient was missing more than seven of his or her natural teeth, they were excluded from the study. Consent was provided at time of arrival to clinic. Spanish-speaking patients who met study criteria completed the surveys that were translated to their native language. In person interviews were conducted in either English or Spanish to screen participants.

### Data collection

Data was collected over the time span of one month (29 days) from June to July 2014. Upon receiving consent, patients were administered a survey in either English or Spanish. The survey asked participants to report various demographic and health information as reported in Table 1 and Table 2. In addition, participants were asked to self-report symptoms associated with bruxism, including teeth grinding or clenching during the day or night, morning jaw soreness, tooth and/or gum soreness, and presence and frequency of headaches. HIV positive participants were asked to report how long they had been treated with HAART. Following completion of the survey, patients proceeded with their regular dental care visit, and clinicians utilized an assessment tool developed based on the study conducted by Ekfeldt, Hugoson, Bergendal, & Helkimo in 1990.

**Table 1:** Demographics (*N* = 93)

Demographic Variables	HIV + ve (*n* = 60)	HIV-ve (*n* = 33)
	n (%)	n (%)
Gender		
Male	56 (93)	16 (48)
Female	3 (5)	17 (52)
Age [mean (SD)]	50 (10)	48 (18)
Smoking	23 (38)	7 (21)
Alcohol Use (> 7 bev./wk)	9 (15)	7 (21)
Reported Recreational Drug Use	9 (15)	2 (6)

**Table 2:** Clinical Variables Among HIV+ and HIV- Participants

Clinical Variables	HIV + ve (*n* = 60)	HIV-ve (*n* = 33)	p*
	n (%)	n (%)	
Temporomandibular Joint Disorder Diagnosis	6 (10)	1 (3)	0.223
Bite Guard Therapy	7 (12)	1 (3)	0.155
Clenching/Grinding:			
Day	29 (48)	8 (24)	0.023
Night	23 (38)	5 (15)	0.020
Teeth/Gum Soreness	25 (42)	5 (15)	0.009
Jaw Soreness	21 (35)	5 (15)	0.041
Headaches	15 (25)	4 (12)	0.141
Psychiatric Disorder	30 (50)	7 (21)	0.007
Tooth Wear	55 (92)	22 (67)	0.002

* *p*-value from Chi-squared test

### Clinical exam

The dentist first recorded the total number of teeth present and then the severity of enamel erosion on each tooth was recorded using the assessment tool. Level of enamel erosion was documented: the number of teeth that had no obvious wear of enamel (Level 0 Erosion); the number of teeth that had wear through the enamel to the dentin in single spots (Level 1 Erosion); the number of teeth that had wear of the dentin up to one-third of the crown height (Level 2 Erosion); and the number of teeth that had wear of the dentin up to more than one-third of the crown height, excessive wear of tooth restorative material or dental material in the crown and bridgework (Level 3 Erosion).

Participants were sorted into three age cohorts: 18–40, 41–65, and 66 and older. Separation into age cohorts helped control for the effect of age on natural enamel erosion and tooth wear. The proportion of participants with teeth that had Level 1, Level 2, and Level 3 enamel erosion was determined. Within each age cohort, the proportion of Level 1, 2, and 3 enamel erosion in HIV patients treated with antiretroviral therapy was compared to a control group of non-HIV patients who were not treated with antiretroviral therapy.

Individual tooth wear index was computed via methods founded by Ekfeldt et al. [7]. This tooth wear index is well reported in the literature as a reliable and clinically valid formula for calculating the degree of tooth wear (Koyano et al., 1990; [4]). This index has been shown to be significantly correlated with bruxism [9]. The following tool allows for indexes to be calculated without influence by amount of tooth loss:

$$ {\mathrm{I}}_{\mathrm{A}}=\left(10\times {\mathrm{G}}_1+30\times {\mathrm{G}}_2+100\times {\mathrm{G}}_3\right)/\left({\mathrm{G}}_0+{\mathrm{G}}_1+{\mathrm{G}}_2+{\mathrm{G}}_3\right) $$

where I_A_ is individual tooth-wear index and G_0_, G_1_, G_2_ and G_3_ are the number of teeth with scores of 0, 1, 2 and 3, respectively.

### Data analysis

Within each age cohort, we used STATA to conduct an ANOVA with age group as a factor and correct for potential confounders, including anxiety, depression, autism, schizophrenia, ADHD (attention deficit hyperactivity disorder), OCD (obsessive compulsive disorder), bipolar disorder or other psychiatric and psychological disorders. As an observational study, a sample size calculation is not applicable. In addition, descriptive statistics and tests of association (including t-tests, various cross-tabulations, Pearson Chi-Square tests) were conducted using SPSS version 24.

## Results

We examined 104 participants in total. A total of 11 participants did not receive wear scores due to not meeting criteria (7 HIV + ve, 4 HIV-ve) and were excluded from all analyses, resulting in a final sample size of 93, of whom 60 were HIV positive (65%) and 33 were HIV negative (35%). A majority of HIV + ve participants were male (93%). A greater proportion of HIV + ve subjects compared to HIV-ve subjects reported the use of tobacco cigarettes and recreational drugs. Those with HIV also had a greater mean age than those without HIV. However, this age difference was 2 years (50 vs. 48, respectively) (Table 1). All 60 HIV + ve subjects were on at least one antiretroviral medication.

Additional clinical variables were measured between HIV + ve and HIV-ve, including presence of temporomandibular disorder (TMD), use of night guard therapy, clenching/grinding during day and at night, teeth/gum soreness, jaw soreness, headaches, psychiatric disorder, and tooth wear (Table 2). A significantly greater proportion of HIV + ve subjects clenched during the day, clenched during night, experienced teeth/gum and jaw soreness, tooth wear, as well as some form of psychiatric disorder when compared to HIV-ve subjects (*p* < 0.05). Although statistically insignificant, a greater proportion of HIV + ve individuals than HIV-ve individuals reported TMD, bite guard therapy and headaches.

**Fig. 1:**
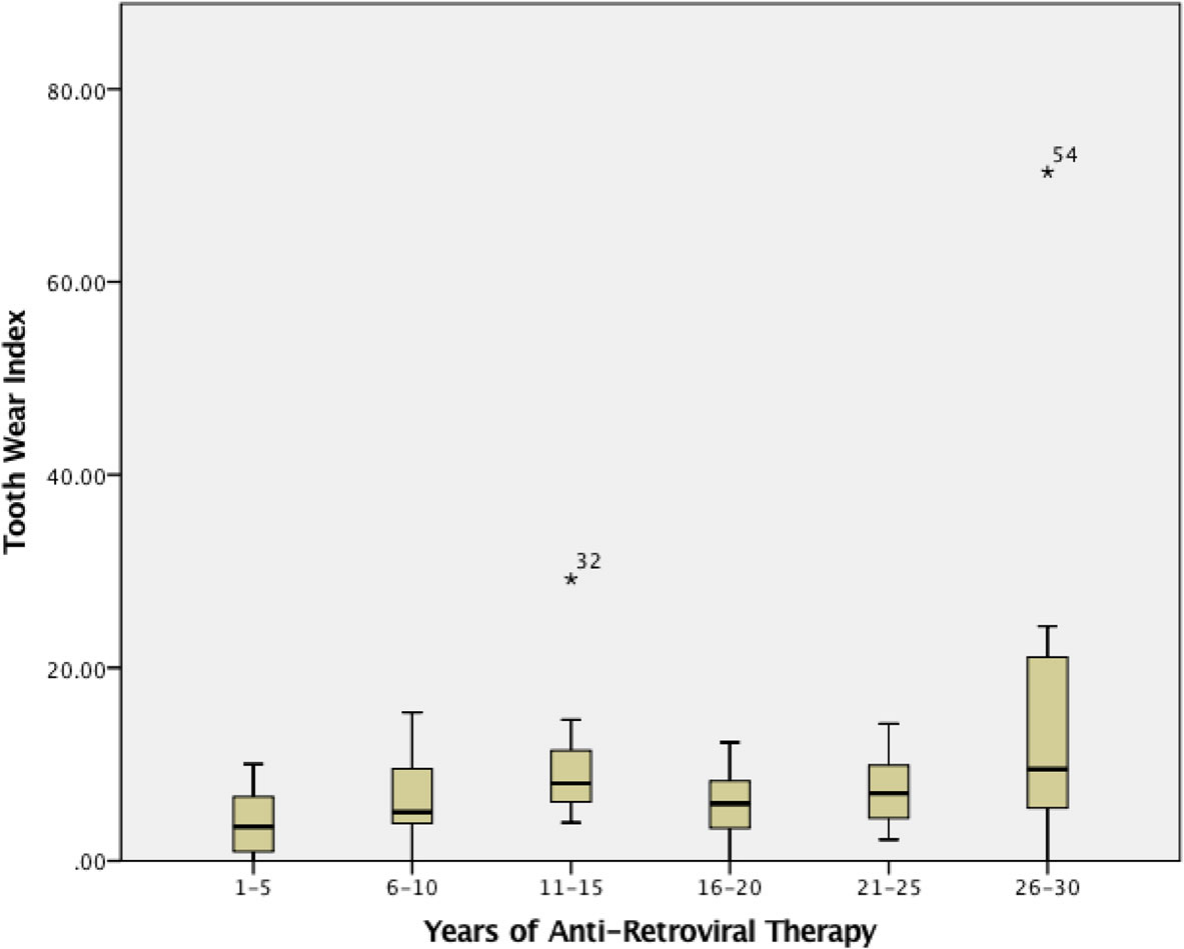
Boxplot of Tooth Wear Index and Anti-Retroviral Therapy Duration (n_1-5yrs_ = 8, n_6-10yrs_ = 14, n_11-15yrs_ = 10, n_16-20yrs_ = 12, n_21-25yrs_ = 7, n_26-30yrs_ = 7)

The presence of tooth wear and mean tooth wear index was calculated and compared between HIV + ve and HIV-ve groups. As seen in Tables 2, 92 and 67% participants of the HIV + ve and HIV-ve groups, respectively, presented with tooth wear (Level 1, 2, or 3). The mean tooth wear index of HIV + ve subjects was higher than the non-HIV infected subjects, though this finding is statistically insignificant (Table 3). However, this demonstrates that among the HIV + ve subjects, there was an average greater number of teeth that presented with tooth wear, or an average lower total number of teeth with wear but with a greater degree of tooth wear on each tooth. Mean tooth wear was also determined within the HIV + ve group and compared between the years of antiretroviral therapy use (1–5 years, 6–10 years, 11–15 years, 16–20 years, 21–25 years, and 26–30 years). As years of antiretroviral therapy use increased, there was an increase in mean tooth wear (Fig. 1).

**Table 3:** Mean Tooth Wear Index by Presence or Absence of Characteristics (N = 93)

Characteristics	Tooth Wear Index (s.d.)	*p*-value (two tailed t-test unequal variances)
HIV		
+	8.2 (10.0)	0.216
-	7.8 (8.3)	
Smoking		
+	6.5 (6.6)	0.530
-	7.7 (10.8)	
Temporomandibular Joint Disorder		
+	6.1 (4.2)	0.506
-	7.4 (9.8)	
Bite Guard Therapy		
+	15.8 (22.8)	0.291
-	6.5 (6.9)	
Clenching-Day		
+	10.6 (12.8)	0.018*
-	5.2 (5.5)	
Clenching-Night		
+	10.6 (13.9)	0.101
-	6.0 (6.4)	
Psychiatric Disorder		
+	8.5 (11.5)	0.394
-	6.6 (7.9)	
Anti-Retroviral Therapy		
+	8.2 (10.0)	0.216
-	5.8 (8.3)	
Sex		
Male	8.4 (10.2)	0.005*
Female	3.5 (5.4)	
Jaw Soreness		
+	8.6 (7.1)	0.348
-	6.8 (10.3)	
Headaches		
+	10.3 (8.2)	0.098
-	6.6 (9.7)	
Teeth/Gum Soreness		
+	10.0 (13.3)	0.130
-	6.0 (6.7)	
7 or more alcoholic beverage per week		
+	4.4 (4.1)	0.024*
-	8.0 (10.1)	

*p-value< 0.05

All participants were categorized by their characteristics and mean tooth wear index was calculated for each characteristic. Multiple independent samples t-tests produced the following results (Table 3). Those who reported clenching during the day presented with nearly twice as much tooth wear than those who reported no clenching (*p* < 0.05). Male subjects tended to have nearly three times as much tooth wear as female subjects (*p* < 0.01). Interestingly, those who reported drinking 7 or more alcoholic beverages per week presented with half as much tooth wear than those who consumed less alcohol (p < 0.05). Though statistically insignificant, other notable findings include a larger mean tooth wear index among those reporting clenching at night, bite guard therapy, headaches, teeth/gum soreness, and retroviral therapy.

### Regression analysis

Following linear regression analysis, the results of Table 4 were produced. HIV + ve status is positively correlated with tooth wear index* (R^2^ = 0.06, *p*-value = 0.017). Both HIV + ve status and age are positively correlated to tooth wear index (R^2^ = 0.13, p-value = 0.002). After accounting for age, a significant, positive correlation was found between HIV presence and tooth wear index (B = 0.71,*p* = 0.025). Age was positively correlated with years of anti-retroviral therapy (*n* = 58, R^2^ = 0.27, *p* < 0.001). This decrease in sample size from 60 was due to those subjects who did not report the duration of anti-retroviral therapy use. Age alone is also positively correlated to tooth wear index (R^2^ = 0.08, p-value = 0.006). The number of years on anti-retroviral therapy alone is positively correlated with tooth wear index (R^2^ = 0.116, *p* = 0.009). Together, age and years of anti-retroviral therapy explained about 13% of the variability in tooth wear (R^2^ = 0.13, *p* = 0.021).

**Table 4:** Summary of Regression Analyses for Predicting Tooth Wear Index Among HIV + ve Individuals

Variable (predictors)	Tooth Wear (Square Root)				
	B	Std. Error B	β	R^2^	p
Age	0.033	0.012	0.284	0.081	0.006**
Years of ART	0.060	0.022	0.341	0.116	0.009**
Both Age and Years of ART				0.131	0.021*
Years of ART	0.047	0.026	0.267		0.076
Age	0.021	0.022	0.142		0.340
HIV + ve	0.780	0.322	0.246	0.061	0.017*
Both HIV + ve and Age				0.131	0.002**
HIV + ve	0.714	0.312	0.225		0.025*
Age	0.031	0.011	0.266		0.008**

*p-value< 0.05

**p-value< 0.01

After controlling for age, we observed that the years of anti-retroviral therapy use was positively correlated with tooth wear index (B = 0.05, p-value = 0.076) (Table 4). The standardization of the tooth wear index as variables demonstrated that a five year increase in years of antiretroviral therapy was associated with a 0.154 standard deviation increase in tooth wear index after adjusting for age (*p* = 0.076). Due to the small sample size, this value may bear significance. However, this finding warrants further investigation in this area with a larger sample size. Notably, the standardized coefficient (beta) for years of retroviral therapy is greater than the standardized coefficient for age (0.267 vs. 0.142). This observation suggests that the years of antiretroviral therapy had a greater effect on tooth wear index than age.

*The transformation to square root of tooth wear index linearized the regression most effectively than tooth wear index, tooth wear index squared and log of tooth wear index. The log of tooth wear index excluded those with tooth wear indexes of 0, thus we utilized the square root of tooth wear index transformation for data analysis and interpretation.

## Discussion

Nearly 90% of HIV + ve subjects taking antiretroviral medications presented with tooth wear. The prevalence of tooth wear among HIV positive individuals found in our study is much greater than that reported by Juvino et al. [11]. This difference may be attributed to our study’s use of the mean tooth wear index formula, whereas Juvino et al. diagnosed bruxism based on a self-reported questionnaire of bruxism behaviors. As suggested by Ekfeldt et al. [7], the tooth wear index formula our study utilized is a reliable indicator of bruxism. Our findings here suggest bruxism and the corresponding tooth wear is a greater problem in PWLHA than previously thought.

As supported by the literature [2, 32], with nearly half of the HIV positive population presenting with a psychiatric disorder, there is a significantly greater prevalence of psychiatric disorders in PLWHA than non-infected subjects in our study. Many of the subjects reported taking psychiatric medications such as Celexa, Cymbalta, Lexapro and Ativan. These medications are used to treat depression and anxiety, which has been determined to be a common psychiatric disorder affecting this population [2, 32]. Though we did not attempt to measure the proportion with anxiety and/or depression within HIV + ve and HIV-ve subject groups, our findings suggest this population is more affected by psychiatric disorders than non-infected individuals. Furthermore, those with HIV presented with both psychiatric disorders and tooth wear, which agrees with the findings of Piccoli et al. [23], who suggested that individuals with depression and anxiety have increased tooth wear.

Our study is the first to investigate the relationship between tooth wear and antiretroviral therapy. Our findings suggest that the longer an individual is on antiretroviral medication, the greater amount of tooth wear he or she will experience. This finding can help to explain the negative effects that antiretroviral medications, as well as psychiatric medications, can have on oral health [10, 24, 31, 33]. However, our findings cannot conclude with certainty that these medications have a direct effect on parafunctional oral habits. It remains challenging to conclude whether age was a major contributing factor to the amount of tooth wear we observed, as Bernhardt et al. [4] demonstrated that tooth wear is natural result of the aging process. We can draw from these findings that PLWHA clench and grind their teeth during both day and night in a much higher proportion and present with a mean tooth wear index about 50% more than those who do not have HIV. Another interesting finding was that the HIV + ve subjects were 95% male. This may be yet another contributing factor to the greater mean tooth wear observed in the HIV + ve subject group. As Bernhardt et al. [4] as suggested, men are more likely to exhibit bruxist behavior, therefore experiencing greater amounts of tooth wear.

Similar to the study by Meless et al. [20], the findings from our study also suggest that knowledge of tooth wear plays a significant role in the comprehensive care of HIV-infected individuals. We must also consider the form of medication during treatment plan discussions for its age appropriateness. For instance, liquid medications often administered to children have cariogenic potential due to the acidity and sugar content [24, 31]. These are a few of the many other factors medical providers must consider when establishing a comprehensive treatment plan for HIV patients to prevent unnecessary oral health problems.

All clinicians should be aware that SSRI antidepressants may cause bruxism or increase the intensity of symptoms. However, SSRIs are not the only medications linked to bruxism. Buspirone, an anxiolytic, is a receptor agonist that increases dopaminergic neuron firing in the ventral tegmental area and increases the synaptic release of dopamine in the prefrontal cortex [9]. These effects ameliorate drug-induced bruxism properties of SSRIs [9]. This finding may be the foundation for future studies on the treatments of these side effects. Importantly, because medical providers have been successful at treating the effects of SSRI-related bruxism [22], an interprofessional intervention between medical and dental providers may be the new way to approach a debilitating oral health care problem and improve health outcomes for PLWHA.

There are a few limitations to our study. Due to the small sample size, our findings cannot be generalized to all population groups. The findings of this study, however, do lay the foundation for further investigation on these populations. Because our sample was taken from a lower socioeconomic group in a community dental clinic, our sample of participants consists of individuals who are typically at a higher risk for developing dental problems, such as periodontal disease, caries, and enamel erosion. Though reliable, the tooth wear index formula is only one clinical measure of bruxism. There are various other intraoral devices that may be more accurate in assessing the degree of bruxism. Future research in this area can benefit from use of such devices.

## Conclusions

The findings of our study demonstrate the importance of oral health side effects associated with the treatment of PLWHA. In patients with already compromised immune systems, chronic bruxism can have a significant impact on HIV positive patients’ oral health and their quality of life. Although research in this area is in its beginning phase, our hope is to initiate a dialogue with medical and dental providers who care for HIV patients. The addition of a medical intervention may compliment and support successful prevention and treatment of bruxism. By educating providers about the potential harmful impact of HIV medications on patients’ dentition and oral health, we can hopefully intervene with night guard therapy at the time a patient is diagnosed with HIV. By prescribing night guard therapy at the time of diagnosis, PLWHA patients will be better protected from the many negative effects associated with chronic bruxism, including temporomandibular disorder, headache, enamel erosion, and tooth wear. The integration of patients’ systemic and oral health care needs will provide more comprehensive treatment for our patients and provide them with a higher quality of life.

## Data Availability

The datasets used and/or analyzed during the study are available from the corresponding author on reasonable request.
